# Characterization of SCC*mec* Instability in Methicillin-Resistant Staphylococcus aureus Affecting Adjacent Chromosomal Regions, Including the Gene for Staphylococcal Protein A (*spa*)

**DOI:** 10.1128/aac.02374-21

**Published:** 2022-03-07

**Authors:** C. R. Scharn, I. A. Tickler, F. C. Tenover, R. V. Goering

**Affiliations:** a Department of Medical Microbiology and Immunology, Creighton University School of Medicine, Omaha, Nebraska, USA; b Cepheid, Sunnyvale, California, USA

**Keywords:** MRSA, SCC*mec*, molecular typing, molecular diagnostics

## Abstract

Staphylococcal cassette chromosome *mec* (SCC*mec*) represents a sequence of clear clinical and diagnostic importance in staphylococci. At a minimum the chromosomal cassette contains the *mecA* gene encoding PBP2a but frequently also includes additional antibiotic resistance genes (e.g., *ermA* and *aadC*; macrolide and aminoglycoside resistance, respectively). Certain regions within SCC*mec* elements are hot spots for sequence instability due to cassette-specific recombinases and a variety of internal mobile elements. SCC*mec* changes may affect not only cassette stability but the integrity of adjacent chromosomal sequences (e.g., the staphylococcal protein A gene; *spa*). We investigated SCC*mec* stability in methicillin-resistant Staphylococcus aureus (MRSA) strains carrying one of four SCC*mec* types cultured in the absence of antimicrobial selection over a 3-month period. SCC*mec* rearrangements were first detected in cefoxitin-susceptible variants after 2 months of passage, and most commonly showed precise excision of the SCC*mec* element. Sequence analysis after 3 months revealed both precise SCC*mec* excision and a variety of SCC*mec* internal deletions, some including extensive adjacent chromosomal loss, including *spa*. No empty cassettes (i.e., loss of just *mecA* from SCC*mec*) were observed among the variants. SCC*mec* stability was influenced both by internal mobile elements (IS*431*) as well as the host cell environment. Genotypically similar clinical isolates with deletions in the *spa* gene were also included for purposes of comparison. The results indicate a role for host-cell influence and the IS*431* element on SCC*mec* stability.

## INTRODUCTION

Methicillin-resistant Staphylococcus aureus (MRSA) continues to be a pathogen of global importance (1). Genes associated with methicillin resistance, primarily *mecA*, are encoded in a family of mobile genetic elements called staphylococcal cassette chromosome *mec* (SCC*mec*) (2). Some SCC*mec* elements also encode other antimicrobial resistance genes such as *ermA* or *aadC* (macrolide and aminoglycoside resistance, respectively). The stable chromosomal location of SCC*mec*, due to specific insertion into the *orfX* locus, has allowed development of a variety of PCR-based diagnostic tests, including rapid detection of MRSA in nasal swabs, blood culture bottles, and wound specimens (3–6). These tests have been used successfully for almost a decade. However, SCC*mec* is a known hot spot for DNA rearrangements, with 14 currently recognized types and numerous subtypes (7). This instability can lead to deletions of a portion or all of the SCC*mec* element, sometimes encompassing adjacent chromosomal sequences (8–14), such as the gene for staphylococcal protein A (*spa*) (15–17) positioned relatively close (e.g., ca. 40 kb) to SCC*mec.* SCC*mec*-associated rearrangements have been primarily described in clinical isolates, presenting challenges to molecular detection. However, little is known regarding the extent to which such events might occur in nonclinical settings, serving as a reservoir of potentially problematic strains upon migration to a clinical environment. We sought to investigate this issue by assessing the frequency and extent to which deletions associated with, but not limited to, SCC*mec* might spontaneously occur over time in different MRSA populations growing in the absence of selection. For purposes of comparison, S. aureus clinical isolates identified as *mecA*-negative and *spa*-nontypeable were also included in the analysis.

## RESULTS

### Longitudinal SCC*mec* stability.

The stability of the four different SCC*mec* elements during 3 months of serial subculturing at room temperature is shown in Table 1. While strain growth differences in brain heart infusion (BHI) broth may have had a minor influence on the frequency of deletion mutants, resulting in methicillin susceptibility over time (e.g., BK11515; Table 1), this did not appear to be widespread since significant differences between susceptible and resistant organisms were not observed in spectrophotometric growth curve experiments (data not shown). SCC*mec* type I in S. aureus COL was stable (i.e., no variants detected) over the 3-month period. However, when transduced from strain COL to strain RN4220 (i.e., strain CRG2358), all susceptible isolates exhibited extensive SCC*mec* I instability after 3 months of subculture involving deletions within SCC*mec* extending to adjacent chromosomal regions, including *spa*.

**TABLE 1:** Frequency of methicillin-susceptible mutants recovered during serial subculture

MRSA strain	SCC*mec* type	Mutants recovered (%) during:		
		Mo 1	Mo 2	Mo 3
COL	I	0.00	0.00	0.00
CRG2358	I	0.00	0.00	0.71^*a*^
HFH-30820	II	0.00	8.33	12.12^*b*^
BK11515	III	0.00	0.25	0.12^*c*^
FPR3757	IV	0.00	0.53	0.94^*c*^

a 100% SCC*mec* internal deletions extending to the adjacent chromosome. No empty cassette or site-specific total cassette excision detected.

b 42% site-specific total cassette excision, 58% SCC*mec* internal and internal plus adjacent chromosomal deletions. No empty cassettes detected.

c 100% site-specific total cassette excision.

**FIG 1:**
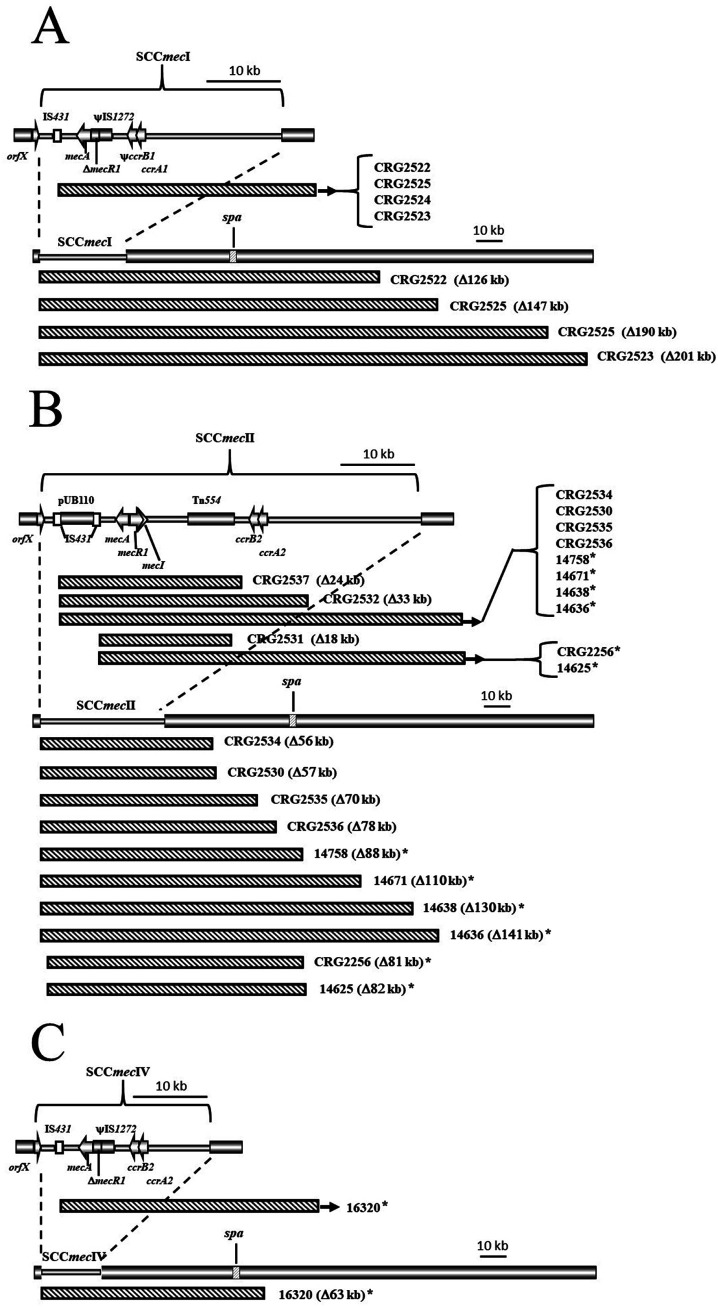
Diagrammatic representation of sequenced deletions (indicated by the pattern bars) associated with SCC*mec* I, II, and IV (A, B, and C, respectively) in S. aureus strains. In each case, SCC*mec* internal deletions are shown in the upper diagram. Instances of deletions including adjacent chromosomal regions are indicated by exit arrows and shown in larger context in the lower diagram. Clinical isolates are indicated with an asterisk.

Strain HRH-30830 (SCC*mec* II) exhibited the highest frequency and greatest variety of susceptibility-associated deletions after 3 months of subculture. PCR testing indicated that 42% of deletions were precise SCC*mec* excision, while the remainder of the isolates showed a mixture of internal SCC*mec* deletions and deletions of SCC*mec* plus adjacent chromosomal sequences (Fig. 1).

PCR analysis confirmed site-specific total SCC*mec* excision in all cefoxitin-susceptible BK11515 (SCC*mec* III) and FPR3757 (SCC*mec* IV) isolates, which was initially detected at 2 months of subculture.

Interestingly, empty cassettes, i.e., the specific loss of only *mecA* from the SCC*mec* element and no other sequences (9), were not detected in cefoxitin-susceptible isolates from any of the SCC*mec* types.

### WGS analysis of SCC*mec*-associated deletion.

Cefoxitin-susceptible colonies derived from CRG2358 and HRH-30830 after 3 months of subculture were shown by PCR analysis to have deletions other than precise SCC*mec* excision. The genomes of these colonies were analyzed by whole-genome sequencing (WGS) (Fig. 1). CRG2358 (SCC*mec* I) derivatives exhibited four deletion patterns (labeled CRG2522, 2523, 2524, and 2525) compared to the parent strain (Fig. 1A). In each case, deletions began at the 3′ end of IS*431*, which is 5′ to *mecA*, and extended approximately 126 kb to 201 kb, including *spa*, which is approximately 40 kb downstream from SCC*mec* I. The deletions observed in HFH-30820 and its derivatives containing SCC*mec* II were more variable, showing eight different patterns (Fig. 1B); however, the deletions always originated at the 3′ end of one of the two IS*431* elements flanking plasmid pUB110 preceding (i.e., 5′ to) *mecA*. In three instances (CRG2531, 2532, and 2537), deletions ranging from 18 kb to 33 kb were internal to SCC*mec* II and included loss of *mecA*. The remaining deletions (CRG2530, 2534, 2535, 2036), which ranged from 56 kb to 78 kb, eliminated SCC*mec* II and adjacent chromosomal regions, the largest of which (CRG2536) came within 7 kb of (but did not include) *spa*.

### WGS analysis of clinical isolates with suspected SCC*mec* deletions.

Seven oxacillin-susceptible S. aureus clinical isolates (Table 2), all of which were negative for *spa* in a commercial assay, had deletions in or around the SCC*mec* II and IV elements, as determined by PCR amplification. WGS analysis (i.e., whole-genome multilocus sequence typing [wgMLST] and traditional MLST) revealed six of the isolates to most likely have previously carried SCC*mec* II (i.e., sequence type 5 [ST5] or ST105 and the presence of pUB110 in two of the isolates; Fig. 1B). Similar to the results found with longitudinal analysis, when mapped against SCC*mec* II strain Mu50, each of these isolates showed deletions originating at the 3′ end of IS*431* either 5′ or 3′ to plasmid pUB110 and extending from 88 kb to 141 kb, always including the remainder of SCC*mec* and *spa* (Fig. 1B). The SCC*mec* IV isolate 16320 (ST8) was mapped to SCC*mec* IV reference strain FPR3757. In comparison to the precise SCC*mec* excision seen in longitudinal analysis, the isolate exhibited extensive deletion (Fig. 1C) also originating at the 3′ end of IS*431*-*mec* and extending to remove the remainder of SCC*mec* and *spa*, ca. 63 kb or 94 kb depending on whether an ancestral arginine catabolic metabolic element (ACME) adjacent to SCC*mec* may have been present.

**TABLE 2:** Bacterial strains

Strain	Relevant characteristics	Reference or source
MRSA stability isolates		
COL	ST5, SCC*mec* I	GenBank accession no. CP000046.1
CRG2358	RN4220, ST8, transduced with SCC*mec* I from strain COL (CP000046.1)	This study
HFH-30820	ST 5, SCC*mec* II	ATCC BP-BAA-1699
BK11515	ST72, SCC*mec* III	Kreiswirth^*a*^
FPR3757	ST8, SCC*mec* IV, ACME	30
*mecA*-*spa*-variant clinical isolates^*b*^		
CRG2256	ST5, SCC*mec* II^*c*^	Iowa
14625	ST105, SCC*mec* II^*c*^	Maryland
14636	ST5, SCC*mec* II^*c*^	Connecticut
14638	ST5, SCC*mec* II^*c*^	Oregon
14671	ST105, SCC*mec* II^*c*^	New York
14758	ST105, SCC*mec* II^*c*^	Kansas
16320	ST8, SCC*mec* IV^*c*^	Smolensk, Russia

a Courtesy of Barry Kreiswirth, Public Health Research Institute, New York.

b Cepheid strain collection.

c Presumed ancestral SCC*mec* type based on ST.

For each of the sequenced isolates (*in vitro* or clinical) deletions specifically began at the 3′ end of an IS*431* element but terminated randomly either within SCC*mec* or in adjacent chromosomal regions (often including *spa*) with no apparent similarity to what might have represented a target sequence.

## DISCUSSION

Since its discovery in 2000 (2), SCC*mec* architecture has been crucial to our understanding of the development and spread of the methicillin resistance gene *mecA* among staphylococcal species, especially S. aureus. Previous studies have reported three different iterations of SCC*mec* rearrangement—precise SCC*mec* excision, empty cassettes (loss only of *mec*A), and partial loss of internal SCC*mec* sequences, which may or may not extend into the adjacent chromosome. This study generated two of the three deletion types, i.e., precise excision of the entire SCC*mec* element with no loss of adjacent chromosomal sequences, and the partial loss of internal SCC*mec* sequence but often including much larger deletions, up to 201 kb. The deletions begin at IS*431* in the SCC*mec* element and extend downstream to include *spa* and other chromosomal sequences. What was not observed among any of the cultured strains or clinical isolates was the empty cassette phenomenon (16). This may be an artifact of the selection of the clinical strains for the study, since they were all suspected of having *spa* deletions, but the lack of empty cassettes among the isolates passaged in the absence of selection was surprising. There is some evidence for the role of antibiotic pressure (i.e., vancomycin) in inducing empty cassettes (9).

Key aspects of past studies have been deletions associated with SCC*mec* II IS*431* elements (18–23). However, to date, questions regarding the role of specific SCC*mec* architecture, other host-cell influence, and the extent to which growth in the absence of selection might relate to such rearrangements have not been examined. As a step toward addressing these issues, we examined the stability of different common SCC*mec* types (I through IV) during 3 months of growth under nonselective conditions. Included were two different strains carrying isogenic SCC*mec* I elements, the first with the element found natively in strain COL and the second when the element was transduced from COL to RN4220 (CRG2358). Of the strains tested, SCC*mec* I in strain COL was the most stable, with no deletions detected after 3 months of passage. However, in the CRG2358 host, deletions in SCC*mec* I were seen at 3 months, all involving extensive loss (i.e., 126 to 201 kb) of adjacent chromosomal sequence including *spa* (Fig. 1A). This implicates as yet unknown host factors in SCC*mec* stability. Host cell influence was also implicated in SCC*mec* IV carriage. This element in FPR3757 exhibited instability at 2 months, which was due to precise excision of SCC*mec*. However, clinical isolate 16320, which is highly related to FPR3757 by conventional and wgMLST, exhibited loss of SCC*mec* and extensive adjacent chromosomal sequence including *spa*. Taken together, these results also point to uncharacterized host cell factors external to but influencing SCC*mec* stability that are deserving of further investigation.

WGS analysis clearly confirmed the influence of IS*431* on SCC*mec* stability. In every case, deletions in susceptible isolates (whether of *in vitro* or clinical origin) began at the 3′ end of the IS*431* elements within SCC*mec*. Deletions of SCC*mec* II showed more variability than SCC*mec* I, perhaps owing to the two IS*431* elements flanking pUB110. However, multiple IS*431* elements alone were not the sole influence, since SCC*mec* III in BK11515 was stable over 3 month’s longitudinal analysis despite containing four IS*431* elements (i.e., flanking internal SCC*mec* and pT181) (24). Factors affecting the extent of IS*431*-associated deletions also remain unclear since termination did not occur in recognizable target sequences. Events such as one-ended transposition, producing identical deletion starting points but asymmetrical terminations in plasmids (25), IS*3*-related “single-ended attack” where a 3′ OH group at a cleaved IS element promotes nucleophilic attack on the opposite end (26), and studies of IS-associated large-scale genomic rearrangements in Gram-negative organisms (27) may hold clues to what was observed here and deserve further investigation. Recombinase genes *ccrA* and *ccrB*, known to be involved in insertion and excision within SCC*mec*, may also play a role here (28, 29).

This study has several limitations. First, this study would have benefited from inclusion of additional SCC*mec* types and host cell backgrounds. In addition, the inclusion and sequence analysis of isolates with empty cassettes may have indicated other rearrangements not previously considered relevant. Nonetheless, our data serve as a proof of principle and provide a sense of the multifactorial nature of SCC*mec* rearrangements. While antibiotic selection may influence SCC*mec* loss and rearrangement (9), study results indicate that cassette-associated changes may frequently occur in nature even in the absence of antimicrobial selection providing a potential reservoir of S. aureus variants. These include precise cassette excision and internal SCC*mec* deletions often extending to the adjacent chromosome. Rates of change were difficult to quantitate due to the influence of host strain and SCC*mec* type. The interrelationship between SCC*mec* architecture and host-cell influence identified here deserves additional investigation to more fully understand the dynamics of SCC*mec* stability and maintenance.

## MATERIALS AND METHODS

### Bacterial strains.

The bacterial strains used in the study are listed in Table 2. For *in vitro* analysis, MRSA strains with SCC*mec* types I through IV were employed. Seven clinical isolates of oxacillin-susceptible S. aureus (six containing SCC*mec* type II and one containing SCC*mec* type IV) from different patients and diverse geographic locations were also studied as examples of naturally occurring deletion mutants.

### Longitudinal serial subculturing.

For SCC*mec* stability studies, MRSA isolates were initially grown on brain heart infusion (BHI) agar with cefoxitin (FOX, 8 μg/mL) to ensure uniform resistance. Isolates were then serially subcultured on BHI agar slants at room temperature every 4 to 5 days and surveyed for resistance stability once a month for a total of 3 months. For each monthly survey, cells were grown overnight in BHI broth (37°C) and serially diluted and plated on BHI agar. After overnight incubation (37°C), the resulting colonies (ca. 300 to 700 per isolate per assay) were replica plated to BHI-FOX plates and again incubated overnight at 37°C. Colonies unable to grow on BHI-FOX (i.e., presumptive methicillin-susceptible) were plated on both BHI and BHI-FOX agar to confirm viability and susceptibility, respectively. Changes in SCC*mec* and adjacent chromosomal regions in confirmed methicillin-susceptible isolates were characterized at the 3-month point as noted below.

### Growth analysis.

MRSA and susceptible derivatives were inoculated into 100 mL of BHI broth in Nephelo culture flasks with a sidearm (Bellco Glass, Vineland, NJ). Bacterial growth was monitored by optical density at 540 nm (OD_540_) absorbance measured every 30 min during 5 h of incubation with shaking at 37°C.

### Molecular characterization.

**(i) PCR and whole-genome sequencing (WGS).** Site-specific excision of the entire SCC*mec* element in methicillin-susceptible variants of SCC*mec* I, II, and III was detected by the presence of a PCR product by utilizing *orfX*-specific forward primers and strain-specific reverse primers targeting chromosomal sequences 3′ to the SCC*mec* element. For strains containing SCC*mec* IV and its derivatives, which may also contain the arginine catabolic metabolic element (ACME) in addition to SCC*mec*, an additional reverse primer in ACME was included to determine the extent of deleted sequences (Table 3). Amplification conditions were as follows: initial denaturation step of 94°C for 2 min, 30 cycles of denaturing at 94°C for 1 min, annealing at 53°C for 1 min, extension at 72°C for 1 min, and a final extension at 72°C for 5 min.

**TABLE 3:** PCR primers for empty-cassette detection

Oligonucleotide	5′–3″ sequence	SCC*mec* type	Reference or source
*orfx*/SCC*mec* junction			
Xsau 325	GGATCAAACGGCCTGCACA	All	31
SCC*mec* and chromosome or ACME junction^*a*^			
A-rev	GAAACTTCATTGGTATATTTAC	I, II, IV	This study
ACME-R	CCTCCTTCACTTAGCACTG	IV	This study
mecIII-R	ACGGTTAGCTTTGGTGGCTT	III	This study

a SCC*mec* IV primers were designed to detect both SCC*mec* IV and SCC*mec* IV-ACME cassette loss.

For WGS, genomic DNA was extracted (DNeasy kit; Qiagen, Germantown, MD), Nextera XT libraries were prepared, and the libraries were sequenced on a MiSeq instrument according to the manufacturer’s instructions (Illumina, San Diego, CA). Sequences were mapped to the following reference strains: for SCC*mec* I, S. aureus strain COL (GenBank accession number CP000046.1), for SCC*mec* II, strain Mu50 (NC_002758.2), and for SCC*mec* IV strain FPR3757 (CP000255) using BioNumerics v.8 (Applied Maths, Belgium) using default settings. No SCC*mec* III-containing isolates were sequenced, because they all were precise excisions of the SCC*mec* element.
